# Bacterial Species Associated with Highly Allergenic Plant Pollen Yield a High Level of Endotoxins and Induce Chemokine and Cytokine Release from Human A549 Cells

**DOI:** 10.1007/s10753-022-01684-3

**Published:** 2022-06-06

**Authors:** Binoy Ambika Manirajan, Ann-Kathrin Hinrichs, Stefan Ratering, Volker Rusch, Andreas Schwiertz, Rita Geissler-Plaum, Gerrit Eichner, Massimiliano Cardinale, Sabine Kuntz, Sylvia Schnell

**Affiliations:** 1 Institute of Applied Microbiology, Research Center for BioSystems, Land Use, and Nutrition (IFZ), Justus-Liebig-University Giessen, 35393 Giessen, Germany; 2 School of Biosciences, Mahatma Gandhi University, Kerala, India; 3 Institut für Integrative Biologie, Stiftung Old Herborn University, Herborn, Germany; 4 MVZ Institut für Mikroökologie GmbH, Herborn, Germany; 5 Mathematical Institute, Justus-Liebig-University Giessen, Giessen, Germany; 6 Department of Biological and Environmental Sciences and Technologies, University of Salento, Lecce, Italy; 7 Institute of Nutritional Sciences, Justus-Liebig-University Giessen, Giessen, Germany

**Keywords:** Plant pollen, Bacteria, Endotoxin, Cytokines, Chemokines, A549 lung epithelial cells

## Abstract

Sensitization to pollen allergens has been increasing in Europe every year. Most studies in this field are related to climate change, phenology, allergens associated with different pollens, and allergic disorders. As a plant microhabitat, pollen is colonized by diverse microorganisms, including endotoxin-producing bacteria which may contribute to pollen allergy (pollinosis). Therefore, bacteria isolated from high allergenic and low allergenic plant pollen, as well as the pollen itself with all microbial inhabitants, were used to assess the effect of the pollen by measuring the endotoxins lipopolysaccharides (LPS) and lipoteichoic acid (LTA) concentrations and their effect on chemokine and cytokine release from transwell cultured epithelial A549 cells as a model of epithelial lung barrier. High allergenic pollen showed a significantly higher level of bacterial endotoxins; interestingly, the endotoxin level found in the bacterial isolates from high allergenic pollen was significantly higher compared to that of bacteria from low allergenic pollen. Moreover, bacterial LPS concentrations across different pollen species positively correlated with the LPS concentration across their corresponding bacterial isolates. Selected bacterial isolates from hazel pollen (HA5, HA13, and HA7) co-cultured with A549 cells induced a potent concentration-dependent release of the chemokine interleukin-8 and monocyte chemotactic protein-1 as well as the cytokine TNF-alpha and interleukin-2 to both apical and basal compartments of the transwell model. This study clearly shows the role of bacteria and bacterial endotoxins in the pollen allergy as well as seasonal allergic rhinitis.

**Supplementary Information:**

The online version contains supplementary material available at 10.1007/s10753-022-01684-3.

## Introduction

Pollen allergy is a major public health problem which has been found regularly increasing [1]. In Europe, the extent of pollen allergy is estimated to be around 40% of the population [2], while the economic impact of allergic diseases is in the range of 55 to 151 billion euros per year [3]. Pollen grains from many plants like grasses, weeds, and trees are recognized as liable for pollinosis [2, 4]. Increased number of pollen count in the environment, changes in the weather, and pollution are the possible reasons for increasing pollinosis incidence [5]. Plant pollen grains carry diverse microbial populations, which include Gram-positive and Gram-negative bacterial as well as fungal species. Furthermore, anemophilous pollen (e.g., birch, mugwort, hazel) carries higher microbial diversity than entomophilous pollen (e.g., winter rapeseed, autumn crocus) [6, 7]. The co-existing microorganisms also have a possible influence in pollen allergy as Colldahl and Nilsson [8] initially reported. Moreover, bacterial endotoxins associated with pollen grains are playing a major role in pollinosis [9].

Endotoxins have been associated to the pathogenesis of a variety of different clinical conditions. The study of endotoxins started in the nineteenth century with Richard Pfeiffer and the term “endotoxin” was given for the heat stable toxin found from heat-inactivated *Vibrio cholerae* [10]. Lipoteichoic acid (LTA) and lipopolysaccharide (LPS) are the major endotoxins synthesized by Gram-positive and Gram-negative bacterial species, and are able to trigger host immune responses [11–13]. LPS consists of a hydrophilic heteropolysaccharide part and a covalently linked hydrophobic lipid portion anchored to the cellular outer membrane. The LPS molecule commonly consists of three structural components: lipid A, a non-repeating core oligosaccharide, and the polysaccharide O-antigen [10, 14, 15]. The hydrophobic region consists of the lipid A portion, which is responsible for the toxic biological effect [16, 17], while the O-antigen is responsible for the immunological response [18]. LTAs are commonly composed of hydrophilic repetitive glycerophosphate units and D-alanine or hexose substituents as well as a lipophilic glycolipid anchor [19, 20]. The glycolipid anchors of LTA are the crucial molecules that trigger the immunity [21]. Airborne antigens such as pollen-associated bacteria interact with or penetrate into the epithelial barrier of the respiratory tract; both innate and adaptive immune responses can be triggered [22, 23]. The first step in this signal cascade is a contact-dependent interaction of molecular patterns such as bacterial LPS or LTA with recognition receptors on cells of the epithelial surface or directly with immune cells [24]. Cell surface bound toll-like receptors (TLRs) as well as C-type lectin receptors (CLRs) detect LPS patterns on Gram-negative bacteria [24–26]. The result of this receptor-ligand crosstalk is a release of pro-inflammatory mediators not only by professional immune cells but also by non-professional immune cells such as epithelial cells. Secreted chemokines such as interleukin-8 (IL8) and monocyte chemotactic protein-1 (MCP-1) as well as cytokines such as tumor necrosis factor alfa (TNF-α) and IL-2 are responsible for the recruitment of granulocytes from the bloodstream to the lung tissue [27], which is a hallmark of an early innate immune response after subjects with asthma or seasonal allergic rhinitis were exposed to allergens [28–30]. Furthermore, in vivo mice studies indicate that exposure to ragweed pollen extract induces a potent TLR4-dependent recruitment of neutrophils and eosinophils by a TLR4-IL-8-receptor-dependent manner [28]. Likewise, it has been shown that stimulation of A549 cells and human asthmatic airway epithelial tissues elicits significant release of TNF-α, IL-4, IL-6, IL-8, IL-9, and IL-13, which in turn stimulates innate and adaptive immune response [31, 32]. The extent of chemokine and/or cytokine secretion, e.g., via the TLR axis, depends on the composition of the bacterial populations on pollen or selected bacterial strains with different LPS-motives [26]. Wind-pollinated plants are mostly the sources of allergenic pollen. The morphological characters of wind-pollinated plant pollen and its exine are different from other types of pollen grains [33, 34]. Our previous studies reported that the bacterial community structure and diversity associated with the pollen microhabitat were clearly affected by the pollination type, as analyzed by both cultivation-dependent and cultivation-independent methods [6, 7].

In this study, we aimed to investigate the possible relationships of bacterial endotoxins associated with both the pollen and the respective bacterial isolates in pollen allergy [6]. The objectives were (I) to compare endotoxin level in pollen grains between five high allergenic and four low allergenic pollen species; (II) to compare the endotoxin level of bacterial isolates from high allergenic and low allergenic pollen species; (III) to correlate the endotoxin levels found in different high allergenic and low allergenic pollen species with those of the bacterial isolates from same pollen species, and (IV) to examine the chemokine and cytokine release from epithelial lung A549 cells after stimulation with selected isolates in a *co*-culture system.

## MATERIALS AND METHODS

### Pollen Sampling

Nine different plants, including five anemophilous high allergenic [birch (*Betula pendula* Roth.), winter rye (*Secale cereale* L.), common hazel (*Corylus avellana* L.), hemp (*Cannabis sativa* L.), and common mugwort (*Artemisia vulgaris* L*.*)] and four entomophilous low allergenic [autumn crocus (*Colchicum autumnale* L.), winter rapeseed (*Brassica napus* L.), blackthorn (*Prunus spinosa* L.), and cherry plum (*Prunus cerasifera* Ehrh.)], were selected for pollen sampling. Flowers were collected from Giessen district (Hessen, Germany) as descripted by Ambika Manirajan et al. [6, 7]. Only the pollen samples from hemp plant species were additionally sampled in spring 2017 and handled as described for the other pollens.

### Isolation of Bacteria and Determination of Colony Forming Units (CFUs) per Gram of Pollen

For the isolation of bacteria, the pollen samples were shaken each in 5 ml of shaking solution (0.05% Tween 80 and 0.18% Na_4_P_2_O_7_) for 30 min and then serially diluted using 0.02% Tween 80 + 0.085% NaCl to a dilution of 10^−5^. This was followed by plating 100 μl of each dilution onto 1:10 diluted AC agar medium (Sigma-Aldrich Chemie GmbH, Steinheim, Germany), as well as on pollen enriched minimal salt medium [6]. The plates were incubated for 5 days at 25 °C in oxic conditions. Total bacterial load was calculated as CFUs per gram dry weight of pollen. CFU results for the pollen of the plants birch, rape, rye, and autumn crocus were taken from Ambika Manirajan et al. [6]. From the CFU counting plates, morphologically different colonies were subcultured from single colonies and pure cultures were isolated as described by Ambika Manirajan et al. [6].

### DNA Extraction and Identification of the Isolates

Genomic DNA of the pure bacterial cultures was extracted using the NucleoSpin DNA isolation kit (MACHEREY NAGEL GmbH & Co. KG, Düren, Germany). Primers EUB9F (5′-GAGTTTGATCMTGGCTCAG-3′) [35] and EUB1492R (5′-ACGGYTACCTTGTTACGACTT-3′) [36] were used to amplify the 16S rRNA gene. PCR products were further purified using the QIAquick PCR purification kit (QIAGEN GmbH, Hilden, Germany) and sequenced by LGC Biosearch Technologies (Berlin, Germany). The high quality region of the 16S rRNA gene sequences was used for comparison with reference sequences by BLAST [37] on the NCBI database and by pairwise alignment on the EzBioCloud [38] database. Bacterial isolates from birch, winter rye, autumn crocus, and winter rapeseed were already reported in our previous publication [6].

### Endotoxin (LPS and LTA) Quantification by Enzyme-Linked Immunosorbent Assay (ELISA)

Suspensions of pollen and Gram-negative and Gram-positive bacteria were used for measuring LPS and LTA concentrations by enzyme-linked immunosorbent assay (ELISA). Therefore, the pollen samples were suspended in sterile pyrogen free water in the proportion of 1 mg ml^−1^, centrifuged and concentrated to OD 50. The bacterial isolates were cultivated in liquid AC medium (1:10), and for harvesting, the bacterial liquid cultures were centrifuged at 4 °C for 30 min at 8000 g. The bacterial pellet of each bacterial isolates was separately collected and the concentration of each isolate’s pellet was adjusted to the OD of 50 using sterile pyrogen free water to adjust for equal concentration. Both the pollen and the bacterial isolate suspensions were diluted to an OD of 12.5 using sterile pyrogen free water to avoid crossing the upper standard endotoxin value in the standard curve. The pollen suspensions were used to determine LPS and LTA concentrations, while the suspensions of Gram-negative bacteria were used to determine LPS concentrations and those of Gram-positive bacteria were used to determine LTA concentrations using Lipopolysaccharides-(LPS)-ELISA kit (Cusabio Biotech, Wuhan, China) and the Lipoteichoic acid-(LTA)-ELISA kit (MyBioSource, San Diego, USA), respectively, following the manufacturer’s instructions. A standard solution of LPS (concentrations of 0 to 400 ng ml^−1^) and LTA (concentrations of 0 to 20 ng ml^−1^) was used to quantify the amounts of each pollen and each bacterial isolate in ng ml^−1^. The LPS-ELISA was performed by adding 100 µl of standards, samples (pollen, Gram-negative bacterial suspension), and blanks to each cavity of the microtiter plate and incubated for 2 h at 37 °C. The LTA-ELISA was performed by adding 50 µl of standards, samples (pollen or Gram-positive isolates suspension), and blanks in each cavity and mixed with 50 µl of Detection A working solution. After incubation for 2 h at 37 °C, the following steps were performed according to the manufacturer’s instructions. Optical density at 450 nm of each well was determined colorimetrically using a microplate reader (Tecan Infinite M200, Männedorf, Schweiz). A four-parameter logistic curve was fitted as standard curve to calculate the concentrations of LPS and LTA. The mean LPS concentrations of pollen as well as Gram-negative and LTA concentrations of pollen as well as Gram-positive bacterial strains were compared between high allergenic and low allergenic plant species.

### Epithelial Cells, Cell Culture Conditions, and Selected Bacterial Isolates

The human epithelial lung cancer cell line A549 (CCL-185) was obtained from American Type Culture Collection (ATCC, Manassas, USA) and a gift from S. Rudloff (https://www.uni-giessen.de/fbz/fsp/meu/methodenplattform). A549 cells were routinely grown in 75-cm^2^ culture flasks using Ham’s F12 K medium (pH 7.4) with 1% GlutaMAX™ and 10% fetal calf serum (Thermo Fisher Scientific, Langenselbold, Germany). Cells were maintained in a humidified atmosphere of 5% CO_2_ in air at 37 °C. Passages (P14-23) were subcultured every 3–4 days before reaching 80–90% confluence, and medium was changed every 2 days.

Selected pollen-associated bacterial isolates from hazel [HA5 (closest relative: *Methylobacterium pseudosasicola*), HA7 (*Spirosoma pollinicola*), and HA13 (closest relative: *Methylobacterium marchantiae*)] (Table S1) were used for *co*-culture studies with A549 cells. Therefore, bacterial isolates were cultured in AC medium (1:10), counted with a Thoma cell counting chamber, and centrifugated at 2778 g for 10 min at room temperature. Supernatants were discarded and bacterial pellets were washed twice with 37 °C warm PBS (pH 7.4). The bacterial pellets were then resuspended in cell culture medium to reach a concentration of 9 × 10^5^ (lowly concentrated) or 9 × 10^7^ (highly concentrated) bacterial cells per 0.33 cm^2^ transwell insert.

### *Co*-culture Incubation Studies with Epithelial A549 Lung Cells

For *co*-incubation studies with the pollen-associated bacterial isolates, pre-confluent A549 cells (60–70%) were trypsinized using a 0.25% (w/v) trypsin/0.53 mM EDTA solution (Thermo Fisher Scientific, Langenselbold, Germany) and 10.000 cells were seeded on transwell inserts with a 0.4 μm pore size polycarbonate membrane (Greiner-Bio-One GmbH, Frickenhausen, Germany). The inserts were placed into a 24-well plate with 0.4-ml medium in the apical and 1.5 ml in the basolateral compartment. Cells were allowed to grow to confluence within 2 days and transepithelial electrical resistance (TEER) was determined before and after the experiments by using a Millicell ERS volt-ohmmeter (Merck Chemicals GmbH, Darmstadt, Germany). TEER readings were taken at 37 °C after equilibrium and wells with TEER values of 125–150 Ohm per 0.33 cm^2^ were used for *co-*incubation studies. Therefore, the medium of the inserts with A549 cells was decanted and the cells were carefully washed with the medium at 37 °C and transferred to the 24-well plate. After washing, two different bacterial concentrations (9 × 10^5^ or 9 × 10^7^) in 0.3-ml culture medium were used for *co-*incubation studies with A549 cells. Therefore, 0.3 ml of each bacterial isolate was added to the apical compartment of the transwell and 1-ml cell medium was applied into the basal compartment. A459 cells were incubated for 5 h at 37 °C, and afterwards, both apical and basal solutions were collected, filtered with a 0.2-µm PES syringe filter (Thermo Fisher Scientific, Langenselbold, Germany), and stored immediately at − 20 °C until determination of cytokine and chemokine concentrations by ELISA.

### A549 Cell Viability

A subset of cultured A549 cells was used for measuring cell counts and cell viability by using the ViaCount™ assay (Luminex BV, MV 's-Hertogenbosch, Netherland). For this, A549 cells were trypsinized from the transwell filter using a 0.25% (w/v) trypsin/0.53 mM EDTA solution before (0 h) and after (5 h) incubation with the bacterial isolates to ensure viability of cells during *co*-cultivation. Twenty microliters of trypsinized cells was incubated with 480 µl ViaCount-reagent™ (Luminex BV, MV 's-Hertogenbosch, Netherland) and incubated for 10 min in the darkness. Immediately after live/dead staining, cells were measured by flow cytometry on the MUSE flow cytometer (Luminex BV, MV 's-Hertogenbosch, Netherland). In all cases, viability remained within 88.7–90.4% and non-viable remained between 10.6 and 12.3%.

### Chemokine and Cytokine Quantification by ELISA

The secretion of chemokines and cytokines by A549 cells into the basal and apical compartments of the transwell system was measured by using ELISA kits for human interleukin-(IL)-8 (Thermo Fisher Scientific, Langenselbold, Germany), human TNF-α (BioCat GmbH, Heidelberg, Germany), human MCP-1, and IL-2 (R&D, Heidelberg, Germany). The ELISAs were performed by using 100 µl of standards, samples (apical and basal), and blanks in each cavity, and after incubation at 37 °C of the indicated times, the following steps were performed according to the manufacturer’s instructions. Finally, optical density (OD) at 450 nm of each cavity was determined colorimetrically using the Synergy H1 microplate reader (BioTEK, Bad Friedrichshall, Germany) and quantification was performed using a standard curve with four-parameter logistic curve fit to calculate the concentrations of the proteins (with Gen5, Version 3.10.). The minimum detectable dose of IL-8, MCP-1, TNF-α, and IL-2 was 1.1, 1.7, 2.0, and 7.0 pg ml^−1^, respectively. All measurements were performed in at least for three independent batches of isolates, with results being reported as mean ± standard deviation (SD).

### Statistical Analysis

**Fig. 1:**
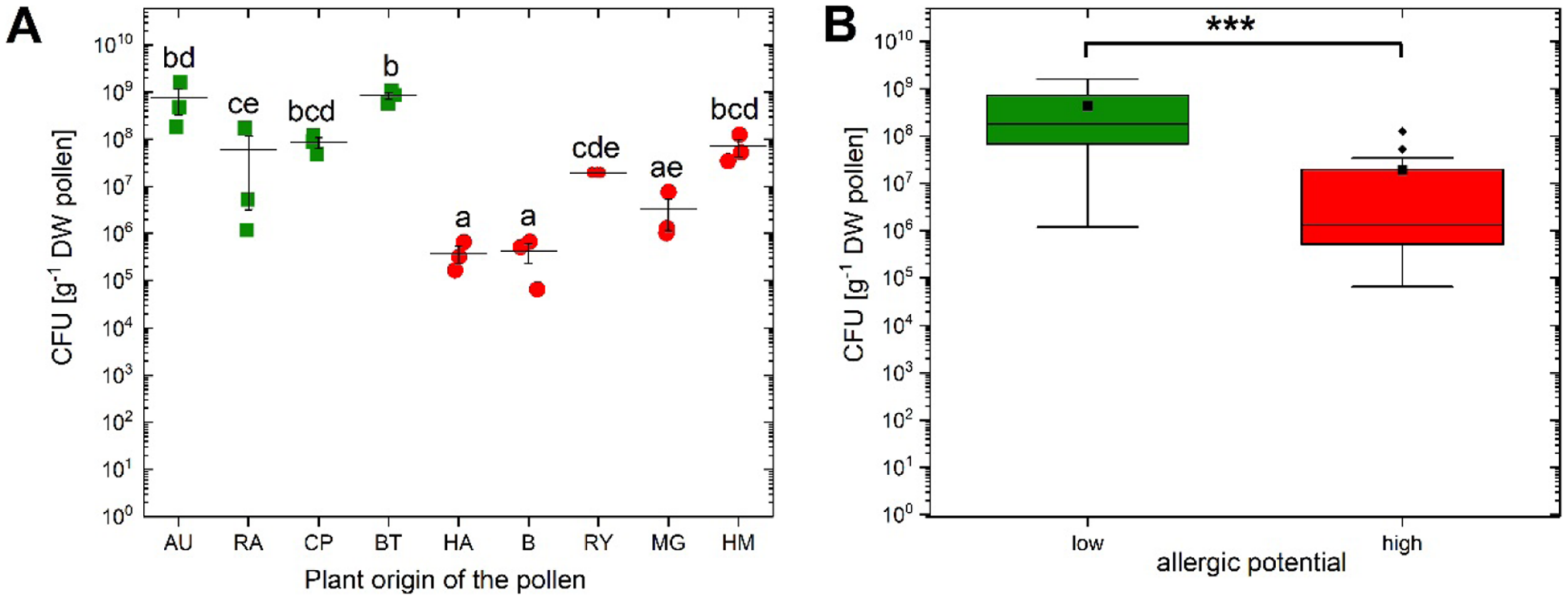
**A** Numbers of cultivable bacteria on flower pollen (CFU g^−1^ DW pollen). ANOVA after log10 transformation (*p* < 0.001) for the factor “Plant origin of the pollen.” Letters above the jittered points indicate statistically significant differences between the numbers of cultivable bacteria (CFU) from various plant pollens at a significance level of *p* < 0.05 (Tukey’s post hoc tests). The horizontal lines showed the mean and the vertical line the standard error; high allergenic pollen species (red circle) and low allergenic pollen species (green square). **B** Box plots (*n* = 12) of cultivable bacteria from plants with high (red color) or low (green color) allergic potential. ***p < 0.001: Wilcoxon’s *p* value for the comparison of low vs. high allergenic potential. B, birch; RY, winter rye; HA, hazel; MG, mugwort; RA, winter rapeseed; AU, autumn crocus; BT, blackthorn; CP, cherry plum; HM, hemp. Box plots (median, box = 25–75% percentiles; mean (black square), outliers (black diamond)). Data of B, RY, HA, and MG from Ambika Manirajan et al. [6].

The results for the ELISA analyses and the CFU counts were statistically analyzed using R (version 4.1.1) [39] with the packages car (version 3.0–11) [40], PMCMRplus (version 1.9.0) [41], and multcomp (version 1.4–17) [42]. The normal distribution was checked with Q–Q (quantile–quantile) plots (Fig. 1 Supplement) of the original data and the log transformed data. LPS-ELISA results with pollen were normally distributed and LTA-ELISA with pollen and CFU counts of the pollen after log transformation. Therefore, a one-way analysis of variance (ANOVA) with Tukey post hoc test was performed for these results. For all comparisons between high and low allergic potential, a two-sided Wilcoxon test was used because the values were not normally distributed. Pearson’s correlation coefficient between mean endotoxin concentrations of the different pollen and mean endotoxin concentrations of all isolates from different pollen species was calculated by means of linear regression using Origin 2017 (Origin Lab Corporation, Northampton, USA). The standard deviations were used as weights. For the results of cytokine and chemokine determination by ELISA, significant differences to controls were analyzed using Prism 9.3.1 (GraphPad, San Diego, USA) using a one-way ANOVA with multiple comparison test (Dunnett). Differences between the normally distributed groups were analyzed using Student’s two-sided paired t-test (*p* < 0.05).

## RESULTS

### Isolation and Characterization of Bacteria from Pollen

A total of 157 morphologically different bacteria were isolated from the pollen of nine different plant species (of which 18 from winter rye, 16 from birch, 15 from winter rapeseed, and 12 from autumn crocus used in this work were already described by Ambika Manirajan et al. [6]) including 62 Gram-positive and 95 Gram-negative isolates (Table S1). The total count of pollen bacterial load ranged from 3.8 × 10^5^ (in hazel) to 8.5 × 10^8^ CFU g^−1^ (in blackthorn) (Fig. 1B). High allergenic pollens (birch, winter rye, mugwort, hemp, and hazel) showed significantly lower CFU numbers than low allergenic plants (winter rapeseed, autumn crocus, cherry plum, and blackthorn) (Wilcoxon test, *p* < 0.001, Table S2). The numbers of CFUs in different plant pollen species were found to be significantly different (ANOVA, *p* < 0.001, Table S2). Tukey’s post hoc test showed significant difference between CFU from nine different plant pollen species (*p* < 0.05). Moreover, hazel and birch had significantly lower CFU counts than all low allergic ones and that autumn crocus and blackthorn have significantly higher CFU counts than the high allergic ones (Fig. 1A, Table S2).

### Determination of Endotoxins (LPS and LTA) in Pollen and Isolates by ELISA

**Fig. 2:**
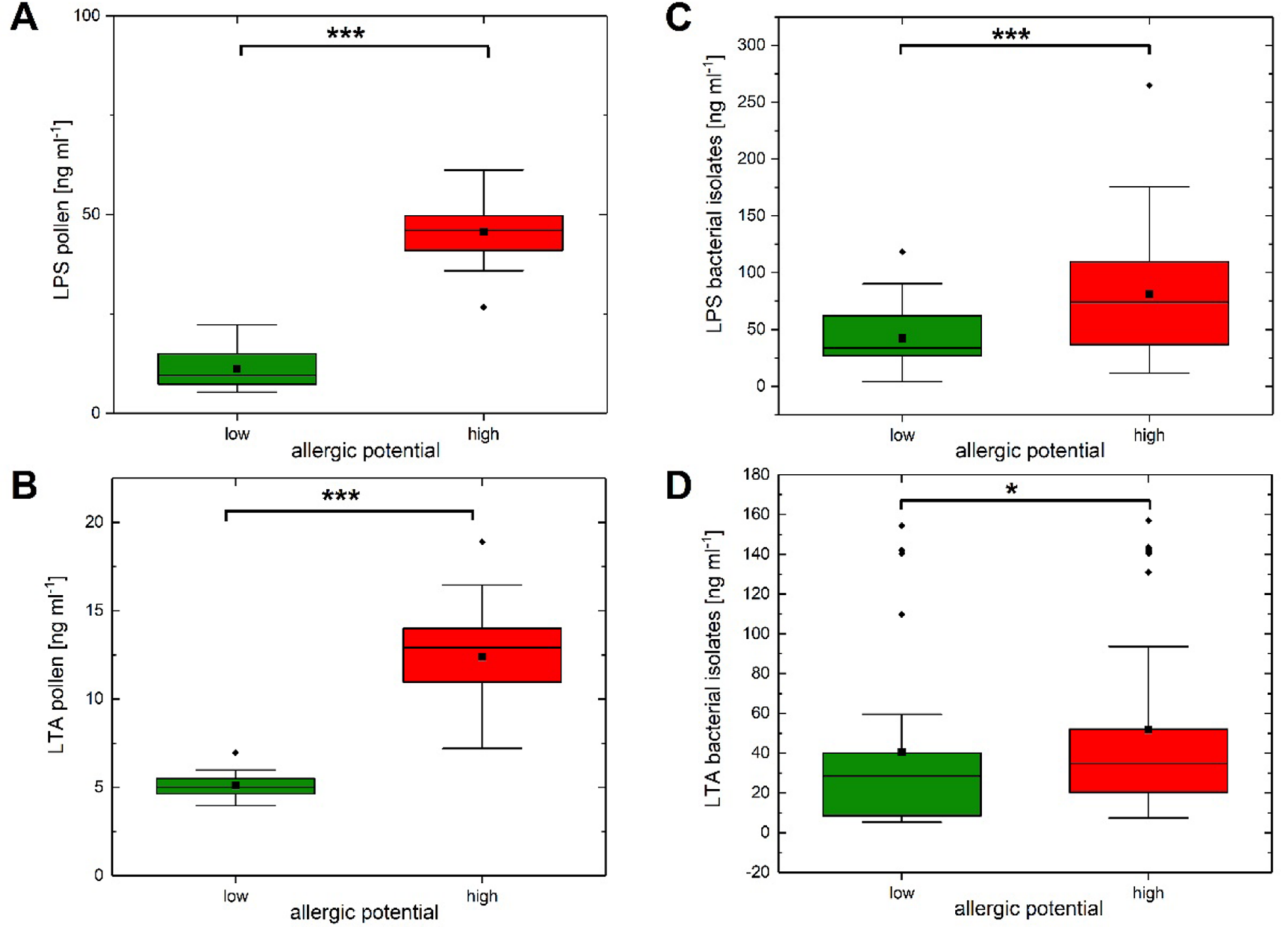
LPS and LTA concentrations in pollen and bacterial isolates measured by ELISA. **A** Box plots of the LPS quantities (ng ml^−1^) in pollen. **B** Box plots of the LTA quantities (ng ml^−1^) in pollen. **C** Box plots of the LPS quantities (ng ml^−1^) in bacterial isolates. **D** Box plots of the LTA quantities (ng ml^−1^) in bacterial isolates. ***p < 0.001, *p < 0.05: Wilcoxon’s *p* values for the comparison of low (green color) vs. high allergenic potential (red color). Box plots (median, box = 25–75% percentiles, mean (black square), outliers (black diamond)).

The results of the ELISA tests for LPS and LTA for the pollen were first pooled for the high and low allergenic plants and then presented as boxplots (Fig. 2). The results of LPS-ELISA of pollen from plants with high and low allergenic potential showed significantly higher LPS concentration in high allergenic pollen samples (Wilcoxon’s *p* < 0.001, Table S2) (Fig. 2A). The same effect can be found for the LTA concentrations (Wilcoxon’s *p* < 0.001, Table S2) (Fig. 2B). The ELISAs were also made with the bacteria isolated from the pollen, and the isolates were sorted according to the presence of LPS or LTA and used for the respective test. The results of the LPS-ELISA of the different bacteria isolated from high and low allergenic pollen samples revealed significantly elevated LPS concentrations in bacterial isolates from high allergenic pollen samples (Wilcoxon’s *p* < 0.001, Table S2) (Fig. 2C). Likewise, the results of the LTA-ELISA were significantly increased in high allergenic pollen samples (Wilcoxon’s *p* < 0.05, Table S2) (Fig. 2D).

**Fig. 3:**
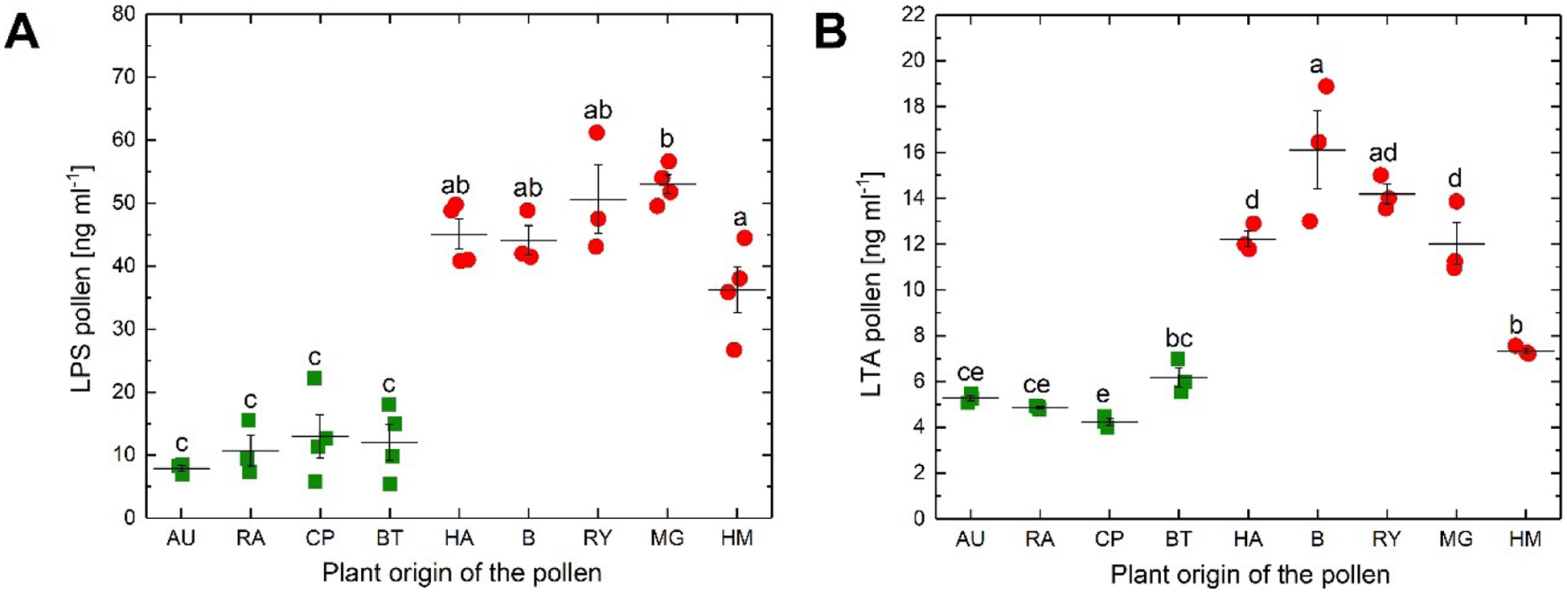
LPS and LTA concentrations in pollen from different plant species measured by ELISA. **A** LPS quantities (ng ml^−1^) and **B** LTA quantities (ng ml^−1^). Letters above the jittered points indicate statistically significant differences between the LPS/LTA from various pollens at a significance level of *p* < 0.05 (Tukey’s post hoc tests). LTA values were log10 transformed before the test. B, birch; RY, winter rye; HA, hazel; MG, mugwort; RA, winter rapeseed; AU, autumn crocus; BT, blackthorn; CP, cherry plum; HM, hemp. The horizontal lines showed the mean and the vertical line the standard error; high allergenic pollen species (red circle) and low allergenic (green square) pollen species.

In addition to the pooled results, the ELISA results of the pollen from each plant were separately analyzed. As expected in view of the pooled results, all pollen from low allergenic plants showed lower LPS values than pollen from high allergenic plants (Fig. 3A). LPS values found from low allergenic plants were not significantly different from each other but significantly different from the values of the high allergic plants (Tukey’s post hoc test, *p* < 0.05). Among the high allergic LPS values, mugwort pollen samples exhibited the highest median LPS quantities and were significantly different from hemp pollen samples that showed a medium level of LPS concentration. But the LPS concentration of hemp pollen samples was not significantly different from other high allergic plants pollen (Tukey’s post hoc test, *p* < 0.05).

For the LTA concentrations (Fig. 3B), the results of the pollen from low allergenic plants pollen samples were similarly low but significantly different (Tukey’s post hoc test, *p* < 0.05). Concentrations of LTA in cherry plum pollen were lowest and not significantly different from autumn crocus and winter rapeseed pollen but significantly different from black thorn pollen, whose concentrations of LTA were not significantly different from autumn crocus and winter rapeseed pollen (Tukey’s post hoc test, *p* < 0.05). Furthermore, LTA concentrations in the group of high allergic plants of birch, hazel, winter rye, and mugwort pollen were not significantly different, but significantly different from the pollen of low allergic plants (Tukey, *p* < 0.05). An exception was hemp whose LTA concentrations of pollen were not significantly different from the concentrations of black thorn, a low allergic plant (Fig. 3B), but were not significantly different from birch, hazel, and winter rye.

### Correlation of Endotoxin Concentration Between Pollen and Isolates

**Fig. 4:**
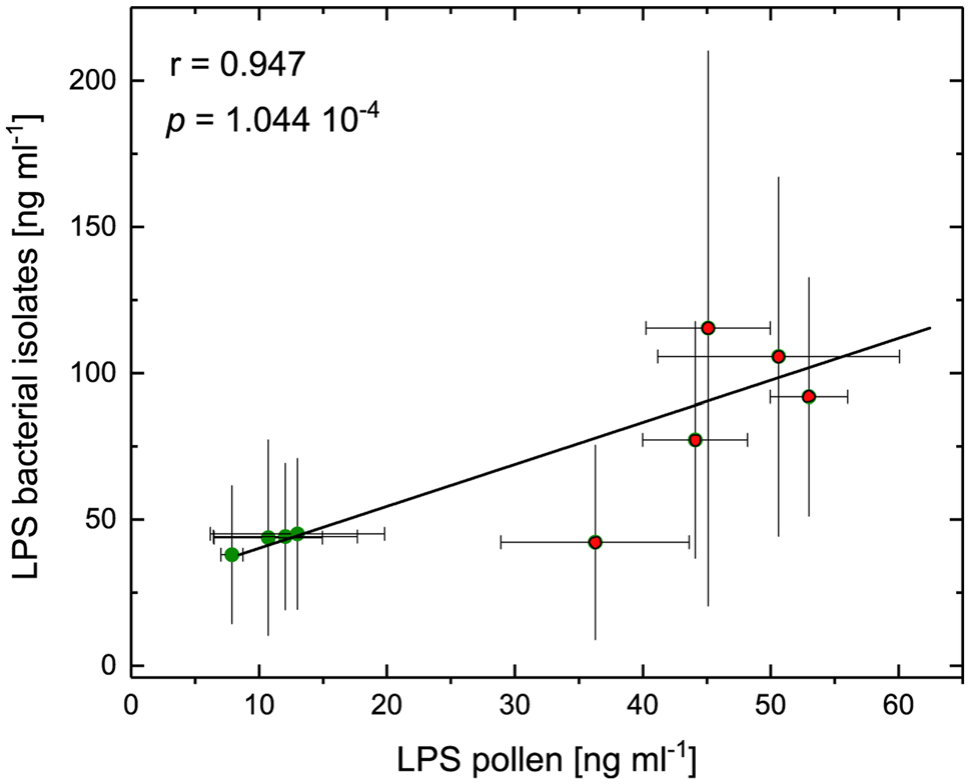
Correlation between LPS concentrations from pollen species and the mean LPS of bacterial isolates from this pollen (Pearson’s *r* = 0.947, *p* = 1.04 x 10^−4^). The straight line shows the linear regression and the error bars show the standard deviation of the means of LTA or LPS concentrations used as weight for the correlation calculations. Symbols: anemophilous high allergenic (red circle); entomophilous low allergenic (green circle).

The LPS concentrations in the nine different pollen species were positively correlated with the mean LPS concentrations of all Gram-negative bacterial isolates from the respective pollen species (Pearson’s correlation coefficient = 0.947, *p* = 1.04 × 10^−4^) (Fig. 4). No correlation was found between LTA concentrations from the different pollen species and the corresponding bacterial isolates analyzed.

### Chemokine and Cytokine Quantitative Determination Assay (ELISA)

**Fig. 5:**
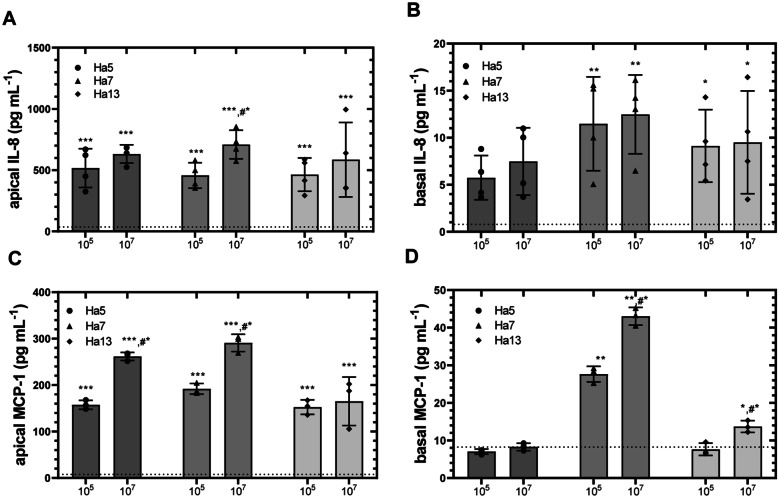
Release of the IL-8 (**A** and **B**) and MCP-1 (**C** and **D**) by A549 cells *co*-cultured with Gram-negative bacteria isolates HA3, HA5, and HA13 in a transwell *co-*culture system. A549 cells cultured on transwell inserts were incubated for 5 h with two different concentrations of bacterial isolates (9 × 10^5^ or 9 × 10^7^ per well). Thereafter, apical and basal supernatants were collected for determination of IL-8 and MCP-1 (pg ml^−1^) using ELISA. Shown are bars with means ± SD (IL-8, *n* = 4; MCP, *n* = 3), one-way ANOVA: *p < 0.05, **p < 0.005, and ***p < 0.001 when compared with controls (dashed line), and t-test: #*p < 0.05 when compared with 10^5^ *co*-incubated bacteria.

In order to investigate whether the high allergenic pollen-associated bacterial isolates HA5 (closest relative: *Methylobacterium pseudosasicola*), HA7 (*Spirosoma pollinicola*), and HA13 (closest relative: *Methylobacterium marchantiae*) might induce chemokine and cytokine release from human epithelial lung cells, these Gram-negative bacterial isolates were *co*-incubated with A549 cells cultured on transwell inserts. Treatment of A549 cells with the isolates induced a significant release of the chemokine IL-8 and MCP-1 into both the apical and–to a lower extent–the basal compartments (Fig. 5). Regarding IL-8, significantly (*p* < 0.001) enhanced concentrations were observed in the apical compartments after incubation of A549 cells with HA5, HA7, and HA13 compared to controls (Fig. 5A). While there is no concentration-dependent effect of HA5 and HA13 on IL-8 secretion by A549 cells, HA7 isolate induced a significantly higher release of IL-8 with 9 × 10^7^ isolates than with 9 × 10^5^ isolates (*p* < 0.01). With regard to the effects of the IL-8 release into the basal compartments, only HA7 and HA13 in both concentrations induced significant effects (*p* < 0.005 and *p* < 0.05). A slight but not significantly enhanced IL-8 release was observed with HA5 (Fig. 5B). Similar to the effects on IL-8 secretion, incubation with the three selected bacterial isolates significantly enhanced MCP-1 release from A549 cells into the apical (Fig. 5C) and basal compartment (Fig. 5D) compared to controls. When A549 cells were cultured in medium alone, MCP-1 levels in apical and basal compartment were 4.9 ± 2.0 and 8.0 ± 1.3 pg ml^−1^ after 5 h, indicating a constitutive secretion of MCP-1. Addition of 9 × 10^5^ bacteria per well significantly induced MCP-1 release into the apical compartment in all cases with HA7 having the most pronounced effect with 191.9 ± 11.6 (*p* < 0.001) followed by HA5 (*p* < 0.001) and HA13 (*p* < 0.001). The release of MCP-1 after addition of 9 × 10^7^ bacteria was further enhanced to 261.7 ± 8.8 (HA5), 290.7 ± 18.5 (HA7), and 165.0 ± 52.1 pg ml^−1^ (HA13) after 5-h incubation. Concerning MCP-1 release, HA5 and HA7 but not HA13 induced a concentration-dependent effect with significantly higher concentrations with 9 × 10^7^ bacteria than with 9 × 10^5^ bacteria (*p* < 0.01). Regarding the effects of the MCP-1 release into the basal compartment, a significantly higher release of MCP-1 by HA7 and HA13 in a concentration-dependent manner was observed (Fig. 5D).

**Fig. 6:**
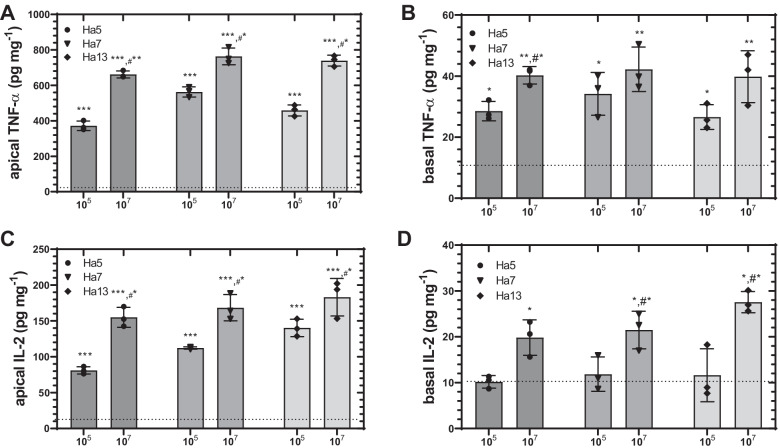
Release of TNF-α (**A** and **B**) and IL-2 (**C** and **D**) by A549 cells *co*-cultured with Gram-negative bacteria isolates HA3, HA5, and HA13 in a transwell system. A549 cells cultured on transwell inserts were incubated for 5 h with different concentrations of bacterial isolates (9 × 10^5^ and 9 × 10^7^ bacteria per well). Thereafter, apical and basal supernatants were collected for determination of TNF-α and IL-2 (pg ml^−1^) using ELISA. Shown are bars with means ± SD (*n* = 3), one-way-ANOVA test: *p < 0.05, **p < 0.005, and ***p < 0.001 when compared with controls (dashed line), and t-test #*p < 0.05 when compared with 9 × 10^5^ *co*-incubated bacteria per well.

Next, the concentration-dependent effect of the three selected bacterial isolates HA5, HA7, and HA13 on cytokine secretion by A549 cells was assayed. Treatment of A549 cells with HA5, HA7, and HA13 induced a significant release of the cytokines TNF-α and IL-2 into both apical and basal compartments (Fig. 6). Concerning TNF-α, a significantly enhanced release from stimulated A549 cells into the apical compartment compared to controls was observed (Fig. 6A). In all cases, concentration-dependent effects were observed with a significantly higher release for 9 × 10^7^ bacterial cells per 0.33 cm^2^ transwell insert than for 9 × 10^5^ bacteria per 0.33 cm^2^ transwell insert (*p* < 0.01). Concerning the effects on TNF-α release into the basal compartment (Fig. 6B), all three bacterial isolates induced an enhanced effect with 9 × 10^5^ bacteria per well insert and to a further extent with 9 × 10^7^ bacteria per well insert (HA5 (*p* < 0.005), HA7 (*p* < 0.005), and HA13 (*p* < 0.01). Similar to the effects on TNF-α secretion, incubation of the three selected bacterial isolates with A549 cells significantly enhanced IL-2 release into the apical (Fig. 6C) and basal compartment (Fig. 6D) compared to controls. In controls, IL-2 concentration in apical compartment was 11.4 ± 1.1 pg ml^−1^ and in the basal compartment 6.5 ± 1.3 pg ml^−1^ after 5 h of *co-*culture, indicating constitutive secretion of IL-2. Incubation with 9 × 10^5^ bacteria per well significantly induced IL-2 concentrations in the apical compartment in all cases (HA7 (*p* < 0.001), HA5 (*p* < 0.001), and HA13 (*p* < 0.001)). The release of IL-2 after addition of 9 × 10^7^ bacteria per well was further enhanced to 155.1 ± 13.9 (HA5), 16,683 ± 18.3 (HA7), and 192.9 ± 9.5 mg ml^−1^ (HA13) after 5-h incubation. Regarding IL-2 release, HA5, HA7, and HA13 induced a concentration-dependent effect with significantly higher release by 9 × 10^7^ bacteria per well than by 9 × 10^5^ bacteria per well (*p* < 0.05). With regard to IL-2 release into the basal compartment, a pattern similar to that observed for the release into the apical compartment was observed with 9 × 10^7^ bacteria per well being more effective than 9 × 10^5^ bacteria per well (Fig. 6D).

## DISCUSSION

In regard to the increasing number of pollen-related allergenic incidence all over the world, subsequent studies about pollen-associated bacteria are of high importance. In these studies, we compare the clinically important endotoxins such as LTA and LPS associated with Gram-positive and Gram-negative bacteria from high allergenic as well as low allergenic pollen habitat.

A mixed microbiota, consisting of Gram-negative and Gram-positive bacteria, contributes to the endotoxin level of the pollen of different plant species. Quantification of endotoxin from the pollen samples and the bacterial isolates from pollen provides basic knowledge about the potential role of pollen-associated bacterial species in pollen allergy, which has received very little attention so far. Endotoxins are the major factors for the pathogenesis of bacterial infection and are known as airborne immunotoxicants for human [43, 44]. Inhalation of the bacterial endotoxin causes blood and lung inflammatory reaction, fever, and shaking chills [45–47]. So far, very little attention has given towards the role of pollen bacterial inhabitance in pollen allergy.

A significant variation of CFU values of nine different pollen species indicates that the species specific structure and allergenic potential of pollen could affect the number of the bacteria inhabiting pollen. The bacterial cultivable fraction in high allergenic pollen species was significantly less compared to low allergenic plants (Fig. 1B). This might be due to morphological difference of exine (tough, resistant outer cuticle of the pollen) between high allergenic and low allergenic pollens. Morphology of pollen and its exine is mainly correlated with the pollination type [48]. Anemophilous pollen grains (e.g., birch, mugwort, hazel) are usually dry, smooth walled, small in diameter, and non-sticky, with limited exine [34, 49, 50]. In contrast, entomophilous pollen grains (e.g., winter rapeseed, autumn crocus) are moist, sticky, and rough, with abundant pollen coat [50–52]. Watson et al. [53] initially reported the positive relationship between endotoxin concentrations and total bacterial counts but here we did not observe the same trend.

The determination of LPS concentrations demonstrated the presence of high levels of LPS in high allergenic pollen species (Figs. 2A and 3A), as expected [46]. Interestingly, also the Gram-negative bacterial species isolated from high allergenic pollen had a higher amount of LPS quantity compared to the isolates from low allergenic pollen species (Fig. 2C). Colldahl and Carlsson [54] first reported that the extracts of bacterial isolates from allergenic pollen induced clinical symptoms (skin reaction test, eye or nasal provocation test) in pollen-sensitive patients. Later, Spiewak et al. [9] showed that the allergenic plant pollen, as well as Gram-negative bacterial isolates from this pollen, contains high LPS level. Recently, Oteros et al. [23] reported that the *Artemisia* (mugwort) pollen acts as vector for airborne bacterial LPS.

Notably, LPS exposure triggers immune responses in bronchial epithelial cells by detection of pathogens through pattern recognition receptors such as TLRs and thus might influence the development of allergic asthma [55, 56]. In our study, we investigated the effect of bacteria isolates HA5, HA7, and HA13 from hazel pollen, on modulating chemokine and cytokine release from lung epithelial A549 cells. These cells were often used as an in vitro model for infection studies because epithelial cells belong to the first cells encountered by inhaled antigens [57–59]. All bacterial isolates induced a potent secretion of chemokines (IL-8 and MCP-1) and cytokines (TNF-α and IL-2) into both apical and–to a lesser extent–basal compartments of the in vitro transwell system (Figs. 5 and 6) indicating that these epithelial cells were able to induce the first step of inflammation. Recently, Kang et al. showed [60] that the stimulation of A549 cells with LPS induces a TLR4-dependent activation of ERK (extracellular signal-regulated kinase) and PI3K/AKT (phosphatidyl-inositol-3-kinase/protein kinase B) both being signaling cascades for the expression of chemokines like IL-8 and MCP-1 [61]. IL-8 and MCP-1 are classical chemokines, which are responsible for the recruitment of granulocytes playing a major role in asthma, allergy, and inflammation [62, 63]. Apart from these observations, we also observed that the hazel bacterial isolates induced the release of TNF-α and IL-2 from A549 cells (Fig. 6). TNF-α belongs to cytokines that increase the expression of cell adhesion molecules on endothelial cells and in this way increase the mobility of immune cells from the bloodstream into the tissue [64, 65]. Together with TNF-α, the cytokine IL-2 was released by A549 cells after exposure with bacterial cells. Although IL-2 is usually secreted by cells of the innate immune system and provides a proliferation signal for lymphocytes, we could show in our study that after stimulation with the bacterial isolate, A549 cells were also able to secrete IL-2. Similar effects of IL-2 release by A549 cells were observed by Holownia et al. [66] using cigarette smoke as an external stimulus.

Interestingly, not all LPS structures seem to have an influence on the mobilization of immune cells. Repo et al. [67] showed that alkali-treated LPS of *Yersinia enteroco litica* had no influence on the expression of adhesion molecules. Neither E-selectin nor the integrin-associated molecules intercellular adhesion molecule-1 (ICAM-1) and vascular cell adhesion molecule-1(VCAM-1) were increased after stimulation with LPS. Given that TNF-α is a strong stimulus [68] for the expression of adhesion molecules and that in our study the isolates HA5, HA7, and HA13 induce the secretion of this cytokine, it seems to be crucial which LPS composition is necessary for this induction. Notably, not only LPS is a potent inducer of cytokines and chemokines release from epithelial cells but also other lipoproteins were able to induce an immune reaction and act with LPS synergistically [69].

Similar to Gram-negative bacteria, Gram-positive bacteria can also trigger the immune response [70]. Gram-positive bacteria associated and colonized with the grass pollen influence the allergic immune responses during skin prick test in human as well as in cell culture [71]. Determination of LTA concentrations of pollen samples revealed the presence of significantly higher level of LTA in high allergenic pollen samples compared to low allergenic pollen samples with both pollen (Fig. 2B) and bacterial isolates (Fig. 2D). However, when looking at the individual results from the pollen of the plants (Fig. 3B), hemp showed a concentration of LTA that is the same as that of the concentration of LTA in the low allergenic plant black thorn. Perhaps, the reason is that hemp is not a typical high allergic plant, which indeed was described as mild to highly allergic plant [72], but cases of allergic reactions due to pollen yet are rare. A few reports of allergic reactions with hemp pollen can be found from areas where hemp occurs natural [73, 74]. 

 Moreover, the LPS quantities between nine different plant pollen species were positively correlated with the average LPS concentration of the bacterial species isolated from respective pollen species. This result indicates that bacterial inhabitants in the pollen habitat contribute to the endotoxin quantity level and are influenced by the factor allergenic potential. The level of endotoxin concentration had a significant correlation with the viable number of Gram-negative bacteria present in water-soluble metalworking fluids [75], dust of livestock barns and poultry houses [76], air of wastewater treatment plants, [77] and surface water [78]. Biologically active lipopolysaccharides associated with dust can induce bronchial inflammation and asthma [79, 80]. Jagielo et al. [81] reported that the concentration of endotoxin in the corn dust strongly influenced the physiologic and biological response in grain dust causing acute airway injury.

These results determined the presence of high concentrations of endotoxins in the pollen of high allergenic plant pollen species. Moreover, bacterial endotoxins from organic and cotton dust are the major causative agents for the development of immune modulatory reactions like bronchial reactivity including fever, asthma, and wheezing [46, 82]. Bacterial endotoxin associated with pollen causes airborne respiratory inflammatory effect [23] and bacterial compounds together with allergens contribute a major role in allergic immune response [83, 84].

In conclusion, the study showed that significantly higher amounts of LPS and LTA occurred in high allergenic pollen in contrast to low allergic pollen and that Gram-negative bacteria isolated from high allergenic pollen contained also a total higher amount of LPS. Moreover, selected Gram-negative bacterial isolates from high allergenic pollen (hazel) induced a potent release of both chemokines and cytokines from epithelial A549 cells, in both apical and basal compartments of the transwell model. All these evidences suggest that Gram-negative bacteria and to a smaller extent Gram-positive bacteria might play an unexpected significant role in pollinosis. Further clinical studies should be performed to confirm the activity of bacteria, LPS, and LTA associated with pollen in pollinosis.

## Supplementary Information

Below is the link to the electronic supplementary material.

Supplementary file1 (DOCX 344 KB)

## Data Availability

Sequences can be found in the NCBI database under the following accession numbers: KX450414-KX450474 and MH813341-MH813436.
