# Supplementary material for: Bacterial Species Associated with Highly Allergenic Plant Pollen Yield a High Level of Endotoxins and Induce Chemokine and Cytokine Release from Human A549 Cells
Source: Inflammation. 2022 Jun 6;45(6):2186–201. doi: 10.1007/s10753-022-01684-3 (PMC9646606; doi:10.1007/s10753-022-01684-3)
Supplement: Supplementary file 1 — Supplementary file1 (DOCX 344 KB) [file 10753_2022_1684_MOESM1_ESM.docx]

**Supplement information**

**Bacterial species associated with highly allergenic plant pollen yield a high level of endotoxins and induce chemokine and cytokine release from human A549 cells.**

Binoy Ambika Manirajan, Ann-Kathrin Hinrichs, Stefan Ratering, Volker Rusch, Andreas Schwiertz, Rita Geissler-Plaum, Gerrit Eichner, Massimiliano Cardinale, Sabine Kuntz, and Sylvia Schnell

Table S1. **Bacterial isolates** cultured from the flower pollen of nine different plant species and identified using the EzTaxon server on basis of 16S rRNA gene sequence for taxonomic affiliation.

| **Name of the isolate(s)** | **Accession number(s) of the 16S rRNA gene(s) sequence(s) of the isolate(s)** | **Winter rye** | **Birch** | **Hazel** | **Mugwort** | **Blackthorn** | **Cherry plum** | **Autumn crocus** | **Winter rapeseed** | **Hemp** | **Taxonomical affiliation (Accession number) [% of pairwise sequence similarity]** |
| --- | --- | --- | --- | --- | --- | --- | --- | --- | --- | --- | --- |
| RYC17 | KX450460 | **✓** |  |  |  |  |  |  |  |  | *Pantoea agglomerans* (AJ233423) [99.7] |
| RYA3, CP19 | KX450447, MH813400 | **✓** |  |  |  |  | **✓** |  |  |  | *Pseudomonas poae* (AJ492829), *Pseudomonas trivialis* (JYLK01000002) [100, 99.9] |
| RYC16 | KX450459 | **✓** |  |  |  |  |  |  |  |  | *Stenotrophomonas rhizophila* (CP007597) [100] |
| RYA10 | KX450454 | **✓** |  |  |  |  |  |  |  |  | *Bacillus simplex* (AB363738) [100] |
| RYA7 | KX450451 | **✓** |  |  |  |  |  |  |  |  | *Exiguobacterium sibiricum* (CP001022) [100] |
| RYB14 | KX450457 | **✓** |  |  |  |  |  |  |  |  | *Pseudarthrobacter defluvil* (AM409361), *Pseudarthrobacter niigatensis* (AB248526) [99.7] |
| RYA9 | KX450453 | **✓** |  |  |  |  |  |  |  |  | *Arthrobacter ginsengisoli* (KF212463), *Arthrobacter humicola* (AB279890), *Arthrobacter oryzae* (AB279889) [99.9] |
| RYB15 | KX450458 | **✓** |  |  |  |  |  |  |  |  | *Nocardioides cavernae* (KX815990) [99.2] |
| RYA2, MG4, CP9 | KX450446, MH813363, MH813390 | **✓** |  |  | **✓** |  | **✓** |  |  |  | *Rathayibacter festucae* (AM410683) [99.7-99.9] |
| RYC18 | KX450461 | **✓** |  |  |  |  |  |  |  |  | *Rhodococcus corynebacterioides* (AF430066) [99.7] |
| RYA5, RYC19 | KX450449, KX450462 | **✓** |  |  |  |  |  |  |  |  | *Rhodococcus fascians* (JMEN01000010) [100] |
| RYA1 | KX450445 | **✓** |  |  |  |  |  |  |  |  | *Streptomyces pratensis* (JQ806215), *Streptomyces anulatus* (DQ026637), *Streptomyces setonii* (MUNB01000146) [100] |
| RYA4, RYA6, Rb-Pol-2, MG8 | KX450448, KX450450, KX450443, MH813366 | **✓** |  |  | **✓** |  |  |  | **✓** |  | *Clavibacter michiganensis* (KF663872, HE608962) [100] |
| RYA11 | KX450455 | **✓** |  |  |  |  |  |  |  |  | *Clavibacter tessellarius* (99.8) [MZMQ01000001] |
| RYA8, Ra-AC-3, HA3, HA4, HA15, HA17, BT9A, BT9B | KX450452, KX450432, MH813343, MH813344, MH813352, MH813353, MH813409, MH813410 | **✓** |  | **✓** |  | **✓** |  |  | **✓** |  | *Frigoribacterium faeni* (Y18807) [99.0-99.9] |
| RYB12, Rb-AC-1 | KX450456, KX450437 | **✓** |  |  |  |  |  |  | **✓** |  | *Exiguobacterium artemiae* (AM072763) [100] |
| Rb-AC-2, Rb-Pol-1 | KX450438, KX450442 |  |  |  |  |  |  |  | **✓** |  | *Pantoea vagans* (EF688012) [100] |
| Ra-Pol-1 | KX450434 |  |  |  |  |  |  |  | **✓** |  | *Pseudomonas endophytica* (LLWHO1000112) [100] |
| Ra-Pol-3, Rb-AC-5, Rb-Pol-3 | KX450436, KX450441, KX450444 |  |  |  |  |  |  |  | **✓** |  | *Rahnella woolbedingensis* (KF308409) [99.9] |
| Rb-AC-3 | KX450439 |  |  |  |  |  |  |  | **✓** |  | *Enterococcus haemoperoxidus* (KB946316), *Enterococcus quebecensis* (GU457262), *Enterococcus silesiacus* (CP013614) [99.9] |
| Ra-AC-4, Rb-AC-4 | KX450433, KX450440 |  |  |  |  |  |  |  | **✓** |  | *Lactococcus garvieae* (AP009332) [100] |
| Ra-Pol-2, Ra-AC-2 | KX450435, KX450431 |  |  |  |  |  |  |  | **✓** |  | *Staphylococcus xylosus* (MRZ001000018) [100] |
| Ra-AC-1, Bb-AC-3 | KX450430, KX450416, |  | **✓** |  |  |  |  |  | **✓** |  | *Rosenbergiella nectarea* (jgi.1084674) [100] |
| AU2, AU5, AU11 | KX450463, KX450464, KX450466 |  |  |  |  |  |  | **✓** |  |  | *Rosenbergiella epipactidis* (KF876184) [99.8-100] |
| Bb-Pol-6 | KX450422 |  | **✓** |  |  |  |  |  |  |  | *Burkholderia andropogonis* (LAQU01000081) [97.9] |
| Bd-AC-3, CAN12, CAN19 | KX450427, MH813427, MH813434 |  | **✓** |  |  |  |  |  |  | **✓** | *Erwinia billingiae* (JN175337) [99.7-100] |
| Bd-AC-4 | KX450428 |  | **✓** |  |  |  |  |  |  |  | *Erwinia tasmaniensis* (CU468135) [99.1] |
| Bd-AC-2 | KX450426 |  | **✓** |  |  |  |  |  |  |  | *Pseudomonas cerasi* (LT222319), *Pseudomonas syringae* (KI657453), *Pseudomonas congelans* (AJ492828), *Pseudomonas ficuserectae* (AB021378) [100] |
| Bd-Pol-1 | KX450429 |  | **✓** |  |  |  |  |  |  |  | *Rahnella aquatilis* (CP003244) [100] |
| Bb-Pol-4 | KX450420 |  | **✓** |  |  |  |  |  |  |  | *Rhizobium soli* (EF363715) [99.7] |
| Bb-AC-4, Bb-Pol-5 | KX450417, KX450421 |  | **✓** |  |  |  |  |  |  |  | *Sodalis praecaptivus* (CP006569) [97.7, 97.8] |
| Bb-Pol-8 | KX450424 |  | **✓** |  |  |  |  |  |  |  | *Staphylococcus cohnii* (D83361) [100] |
| Bd-AC-1 | KX450425 |  | **✓** |  |  |  |  |  |  |  | *Chryseobacterium indoltheticum* (AY468448) [100] |
| Ba-AC-1 | KX450414 |  | **✓** |  |  |  |  |  |  |  | *Hymenobacter roseus* (HG965772) [98.3] |
| Bb-AC-2 | KX450415 |  | **✓** |  |  |  |  |  |  |  | *Microbacterium phyllosphaerae* (AJ277840) [100] |
| Bb-Pol-2 | KX450419 |  | **✓** |  |  |  |  |  |  |  | *Micrococcus yunnanensis* (FJ214355), *Micrococcus luteus* (CP001628) [100] |
| Bb-Pol-1, MG24 | KX450418, MH813381 |  | **✓** |  | **✓** |  |  |  |  |  | *Streptomyces albidoflavus* (Z76676), *Streptomyces violascens* (AY999737), *Streptomyces hydrogenans* (AB184868), *Streptomyces daghestanicus* (DQ442497) [100] |
| AU19, CAN3, AU8, AU14 | KX450474, MH813419, KX450465, KX450469 |  |  |  |  |  |  | **✓** |  | **✓** | *Erwinia persicina* (BCTN01000053) [97.9-99.5] |
| AU17 | KX450472 |  |  |  |  |  |  | **✓** |  |  | *Lonsdalea iberica* (LUTP01000101) [100] |
| AU15 | KX450470 |  |  |  |  |  |  | **✓** |  |  | *Micrococcus yunnanensis* (FJ214355) [99.7] |
| AU12 | KX450467 |  |  |  |  |  |  | **✓** |  |  | *Erwinia piriflorinigrans* (GQ405202) [98.5] |
| AU16 | KX450471 |  |  |  |  |  |  | **✓** |  |  | *Rahnella victoriana* (KF308403), *Rahnella woolbedingensis* (KF308409 [99.9] |
| AU13 | KX450468 |  |  |  |  |  |  | **✓** |  |  | *Brevibacterium frigoritolerans* (AM747813) [100] |
| AU18, HA8, HA11, HA23, CAN1, CAN2, CAN7, CAN8, CAN11, CAN15, CAN20, CAN21 | KX450473, MH813347, MH813350, MH813359, MH813417, MH813418, MH813422, MH813423, MH813426, MH813430, MH813435, MH813436 |  |  | **✓** |  |  |  | **✓** |  | **✓** | *Curtobacterium flaccumfaciens* (AJ312209) [98.6-100] |
| HA1, CP7 | MH813341, MH813388 |  |  | **✓** |  |  | **✓** |  |  |  | *Aureimonas glaciei* (KU253627) [98.8, 99.2] |
| HA2 | MH813342 |  |  | **✓** |  |  |  |  |  |  | *Sphingomonas faeni* (AJ429239) [99.7] |
| Bb-Pol-7, HA5, MG9 | KX450423, MH813345, MH813367 |  | **✓** | **✓** | **✓** |  |  |  |  |  | *Methylobacterium pseudosasicola* (jgi.1071178) [99.3-99.9] |
| HA7 | MH813346 |  |  | **✓** |  |  |  |  |  |  | *Spirosoma pollinicola* (MG589923) [100] |
| HA9A, HA9B | MH813348, MH813349 |  |  | **✓** |  |  |  |  |  |  | *Amnibacterium kyonggiense* (FJ527819) [98.3, 98.5] |
| HA13 | MH813351 |  |  | **✓** |  |  |  |  |  |  | *Methylobacterium marchantiae* (FJ157976) [98.9] |
| HA18 | MH813354 |  |  | **✓** |  |  |  |  |  |  | *Pseudomonas agarici* (AKBQ01000002) [98.6] |
| HA19, BT6, BT7 | MH813355, MH813406, MH813407 |  |  | **✓** |  | **✓** |  |  |  |  | *Frondihabitans peucedani* (FM998017) [98.5, 100] |
| HA21A, HA21B, HA22, MG5, MG18, MG19 | MH813356, MH813357, MH813358, MH813364, MH813375, MH813376 |  |  | **✓** | **✓** |  |  |  |  |  | *Bacillus altitudinis* (ASJC01000029) [99.7-100] |
| HA24 | MH813360 |  |  | **✓** |  |  |  |  |  |  | *Paenibacillus kyungheensis* (KF793934) [98.6] |
| MG1 | MH813361 |  |  |  | **✓** |  |  |  |  |  | *Bacillus subtilis* (AMXN01000021), *Bacillus tequilensis* (AYTO01000043) [99.9] |
| MG2, MG10, MG12, MG16 | MH813362, MH813368, MH813370, MH813373 |  |  |  | **✓** |  |  |  |  |  | *Burkholderia multivorans* (ALIW01000278) [99.9-100] |
| MG6, MG20 | MH813365, MH813377 |  |  |  | **✓** |  |  |  |  |  | *Rhodococcus cerastii* (FR714842) [99.9, 98.5] |
| CP12 | MH813393 |  |  |  |  |  | **✓** |  |  |  | *Rhodococcus sovatensis* (KU189221), *Rhodococcus cerastii* (FR714842) [98.5] |
| MG11, MG17 | MH813369, MH813374 |  |  |  | **✓** |  |  |  |  |  | *Bacillus safensis* (ASJD01000027), *Bacillus zhangzhouensis* (JOTP01000061) [99.9, 100] |
| MG14 | MH813371 |  |  |  | **✓** |  |  |  |  |  | *Streptomyces mexicanus* (AF441168) [99.9] |
| MG15, CP5 | MH813372, MH813386 |  |  |  | **✓** |  | **✓** |  |  |  | *Agreia pratensis* (AJ310412) [99.9, 99.7] |
| MG21 | MH813378 |  |  |  | **✓** |  |  |  |  |  | *Arthrobacter agilis* (X80748) [99.8] |
| MG22 | MH813379 |  |  |  | **✓** |  |  |  |  |  | *Microvirga soli* (KX247636) [99.8] |
| MG23 | MH813380 |  |  |  | **✓** |  |  |  |  |  | *Sphingomonas aerolata* (AJ429240) [99.5] |
| BT1, BT16 | MH813402, MH813416 |  |  |  |  | **✓** |  |  |  |  | *Pseudomonas canadensis*  (AYTD01000015) [99.8] |
| BT2, BT12 | MH813403, MH813413 |  |  |  |  | **✓** |  |  |  |  | *Glutamicibacter bergerei* (AJ609630) [99.8] |
| BT3 | MH813404 |  |  |  |  | **✓** |  |  |  |  | *Microbacterium oxydans* (Y17227), *Microbacterium maritypicum* (AJ853910) [100] |
| BT5, BT15 | MH813405, MH813415 |  |  |  |  | **✓** |  |  |  |  | *Curtobacterium oceanosedimentum*  (EF592577) [99.7] |
| BT8, BT10, BT14 | MH813408, MH813411, MH813414 |  |  |  |  | **✓** |  |  |  |  | *Curtobacterium herbarum* (AJ310413), *Curtobacterium oceanosedimentum*  (EF592577) [98.6] |
| BT11 | MH813412 |  |  |  |  | **✓** |  |  |  |  | *Brevibacterium aurantiacum*  (X76566) [98.7] |
| CP1 | MH813382 |  |  |  |  |  | **✓** |  |  |  | *Methylobacterium bullatum* (GU983169) [99.5] |
| CP2, CP18 | MH813383, MH813399 |  |  |  |  |  | **✓** |  |  |  | *Methylobacterium cerastii* (FR733885) [100, 99.8] |
| CP3 | MH813384 |  |  |  |  |  | **✓** |  |  |  | *Subtercola frigoramans* (AF224723) [97.9] |
| CP4 | MH813385 |  |  |  |  |  | **✓** |  |  |  | *Subtercola boreus* (AF224722) [98.4] |
| CP6, CP13 | MH813387, MH813394 |  |  |  |  |  | **✓** |  |  |  | *Friedmanniella antarctica* (Z78206) [100, 99.8] |
| CP8 | MH813389 |  |  |  |  |  | **✓** |  |  |  | *Amnibacterium soli* (EU432172) [99.8] |
| CP10, CP11 | MH813391, MH813392 |  |  |  |  |  | **✓** |  |  |  | *Williamsia limnetica* (HQ157192) [99.2, 99.1] |
| CP14 | MH813395 |  |  |  |  |  | **✓** |  |  |  | *Kineococcus aurantiacus* (X77958) [99] |
| CP15 | MH813396 |  |  |  |  |  | **✓** |  |  |  | *Nakamurella silvestris* (KP899234) [98.7] |
| CP16, CP17 | MH813397, MH813398 |  |  |  |  |  | **✓** |  |  |  | *Frondihabitans sucicola* (JX876867) [98.5, 98.6] |
| CP20 | MH813401 |  |  |  |  |  | **✓** |  |  |  | *Caballeronia sordidicola* (FCOC01000044) [98.5] |
| CAN4, CAN9, CAN10, CAN18 | MH813420, MH813424, MH813425, MH813433 |  |  |  |  |  |  |  |  | **✓** | *Morganella psychrotolerans* (DQ358135) [98.7] |
| CAN5 | MH813421 |  |  |  |  |  |  |  |  | **✓** | Microbacterium testaceum  (X77445) [99.8] |
| CAN13 | MH813428 |  |  |  |  |  |  |  |  | **✓** | *Pseudomonas coleopterorum* (KM888184), *Pseudomonas rhizosphaerae* (CP009533) [99.8] |
| CAN14 | MH813429 |  |  |  |  |  |  |  |  | **✓** | *Bacillus zhangzhouensis* (JOTP01000061), *Bacillus safensis* (ASJD01000027) [99.8] |
| CAN16 | MH813431 |  |  |  |  |  |  |  |  | **✓** | *Bacillus tequilensis* (AYTO01000043), *Bacillus subtilis* (AMXN01000021) [99.7] |
| CAN17 | MH813432 |  |  |  |  |  |  |  |  | **✓** | *Bacillus aryabhattai* (EF114313) [99.8] |

**Table Supplement 2:** Statistical results of the comparisons of CFU count, LPS ELISA and LTA ELISA between the different pollen species and allergenic potential (mean of all high or low allergic pollen species or bacterial isolates from high or low allergic plants).

| Parameter | Figure | Transformation | Test | p-value |
| --- | --- | --- | --- | --- |
| CFU pollen species | 1A | Log10 | ANOVA | 2.36 × 10^-7^ |
| CFU pollen species | 1A | Log10 | Tukey | see Fig. 1A |
| CFU allergy pollen | 1B | -- | Wilcoxon | 0.00053 |
| LPS allergy pollen | 2A | -- | Wilcoxon | 1.7 × 10^-6^ |
| LTA allergy pollen | 2B | -- | Wilcoxon | 1.13 × 10^-5^ |
| LPS allergy isolates | 2C | -- | Wilcoxon | 0.00042 |
| LTA allergy isolates | 2D | -- | Wilcoxon | 0.0465 |
| LPS pollen species | 3A | -- | ANOVA | 1.43 × 10^-11^ |
| LPS pollen species | 3A |  | Tukey | see Fig. 2C |
| LTA pollen species | 3B | Log10 | ANOVA | 6.63 × 10^-13^ |
| LTA pollen species | 3B | Log10 | Tukey | see Fig. 2D |


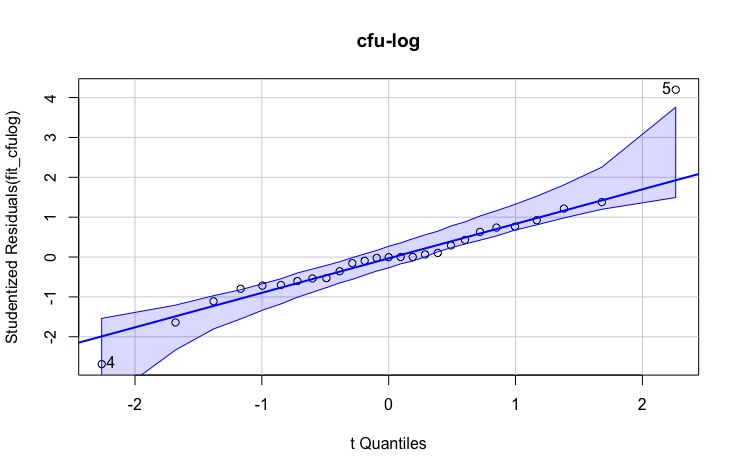

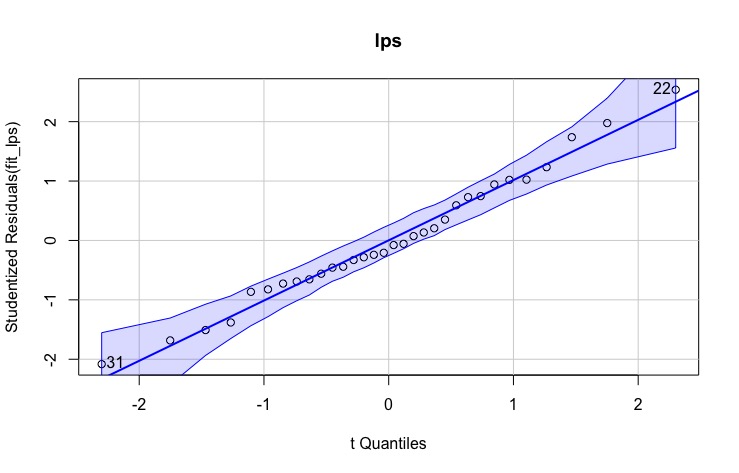


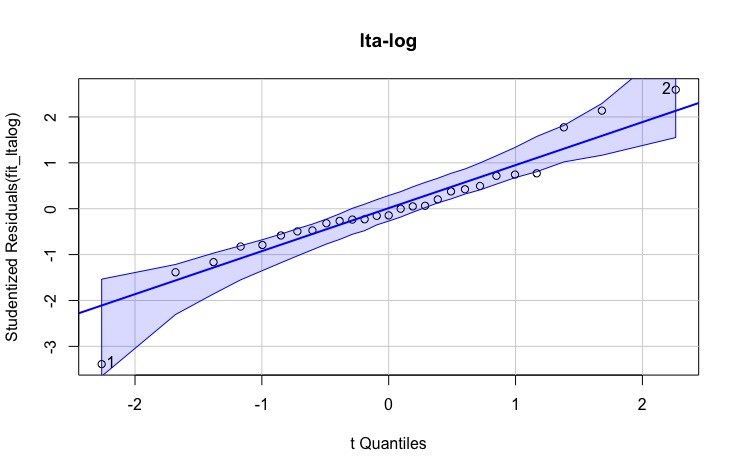


**Figure 1 Supplement**: Quantile-quantile plots of the residuals of the one-factorial ANOVA model fits of the values of the CFU counting (Fig. 1), the LPS ELISA and the LTA ELISA with the single pollen species (Fig. 2). The values for the CFU counting and the LTA ELISA had to be logarithmised to obtain normal distribution.
